# Debris flow volume prediction model based on back propagation neural network optimized by improved whale optimization algorithm

**DOI:** 10.1371/journal.pone.0297380

**Published:** 2024-04-09

**Authors:** Bo Ni, Li Li, Hanjie Lin, Yue Qiang, Hengbin Wu, Zhongxu Zhang, Yi Zhang

**Affiliations:** Department of Civil Engineering, Chongqing Three Gorges University, Wanzhou, 404100, Chongqing, China; Univerzitet Singidunum, SERBIA

## Abstract

Debris flow is a sudden natural disaster in mountainous areas, which seriously threatens the lives and property of nearby residents. Therefore, it is necessary to predict the volume of debris flow accurately and reliably. However, the predictions of back propagation neural networks are unstable and inaccurate due to the limited dataset. In this study, the Cubic map optimizes the initial population position of the whale optimization algorithm. Meanwhile, the adaptive weight adjustment strategy optimizes the weight value in the shrink-wrapping mechanism of the whale optimization algorithm. Then, the improved whale optimization algorithm optimizes the final weights and thresholds in the back propagation neural network. Finally, to verify the performance of the final model, sixty debris flow gullies caused by earthquakes in Longmenshan area are selected as the research objects. Through correlation analysis, 4 main factors affecting the volume of debris flow are determined and inputted into the model for training and prediction. Four methods (support vector machine regression, XGBoost, back propagation neural network optimized by artificial bee colony algorithm, back propagation neural network optimized by grey wolf optimization algorithm) are used to compare the prediction performance and reliability. The results indicate that loose sediments from co-seismic landslides are the most important factor influencing the flow of debris flows in the earthquake area. The mean absolute percentage error, mean absolute error and R^2^ of the final model are 0.193, 29.197 × 10^4^ m^3^ and 0.912, respectively. The final model is more accurate and stable when the dataset is insufficient and under complexity. This is attributed to the optimization of WOA by Cubic map and adaptive weight adjustment. In general, the model of this paper can provide reference for debris flow prevention and machine learning algorithms.

## 1 Introduction

Catastrophic earthquakes usually cause strong disturbances in the bedrock of the slope, which produces a large amount of loose material [1]. Therefore, the large amount of loose material from the Wenchuan earthquake provided excellent material source conditions for debris flows in the study area of this paper [2]. Moreover, debris flow disasters will not only cause damage to roads and bridges, but also lead to river blockage [3–6]. In general, the size of the mudslide volume is a direct characterization of the mudslide hazard [7]. Therefore, reliable and accurate prediction of debris flow volume is of great significance for debris flow prevention and control projects, emergency rescue and disaster relief [8].

The estimation of debris flow volume is mainly divided into two categories. The first category is mainly based on empirical formulas based on actual survey data. For example, Ma, Chang, Cao and Gartner have established different empirical formulas for different study areas [9–12]. The main contributions of the above four researchers are shown in Table 1.

**Table 1:** Main contributions.

Name	Main contributions
Ma	Ma et al. demonstrated that there is a strong correlation between the volume of debris flow in the earthquake area and the quality of loose matter, but the correlation with the basin area is weak. And it appeared that the activity of debris flows will significantly increase in the short term after an earthquake.
Chang	Chang et al. found that watershed area and loose material area have a significant impact on the volume of debris flow. The impact of the geological index on the volume of debris flow depends on the duration of rainfall.
Cao	Cao et al. estimated the volume of debris flows by combining field investigations, optical remote sensing, and interferometric synthetic aperture radar (InSAR). The results indicated that: (1) The quality of sediment is the main influencing factor of debris flow volume. (2) The InSAR technology can continuously monitor debris flows, avoiding the limitations of the other two investigation methods.
Gartner	Gartner et al. established six regression models to predict the volume of debris flows caused by wildfires in different regions and geological conditions. The results indicated that: (1) The total amount of rainstorm and the drainage area of moderate and high burn are the most relevant variables with debris flow volume. (2) The more severely burned the soil, the more susceptible it is to erosion, and soil with burns exceeding 30% is particularly susceptible to erosion.

Table 1 shows that the volume of loose material is the main factor influencing the volume of debris flows. This is particularly evident in the seismic zone. In addition, the basin area and rainstorm are also important factors affecting the volume of debris flow. However, empirical formulas have regional applicability. This means that an empirical formula for one study area may not be applicable to another study area. Moreover, the strong subjectivity of the researchers and the poor logic between the data may lead to large errors in the results of the empirical formula [13]. Therefore, using the traditional empirical formula to estimate the volume of debris flow is unreliable and has poor adaptability.

With the development of machine learning, artificial intelligence algorithms are gradually applied to natural disaster prediction. [14] Therefore, the second prediction method based on machine learning is applied to debris flow events. Zhao used machine learning algorithm and time series algorithm to predict debris flow events in advance [15]. The results show that the Extra Trees (ET) model accurately predicts mudslide events 35 minutes in advance. In addition, Kern said that future work should use machine learning models to improve debris flow volume prediction [16]. Lee used artificial neural network (ANN) to predict the volume of debris flow under extreme rainfall in central South Korea [17]. The prediction results provide a favorable reference for the control design of debris flow-prone areas in South Korea. In general, the machine learning model has good effect and potential in debris flow volume prediction, so more research is needed.

Back propagation neural network (BPNN), as the most widely used and mature neural network model, has been widely used in various fields of civil engineering [18,19]. However, when machine learning algorithms are used to predict geohazard events, the predictions are highly correlated with the quality of the dataset [20]. This is a challenge for neural networks. Because the neural network needs a large enough data set to output reliable and accurate prediction results [21]. As a result, BPNN may output unstable and low-accuracy results when there is insufficient data. However, obtaining data on geohazards through physical surveys would consume a great deal of human and financial resources. At present, optimization algorithms have been shown to improve the prediction accuracy and reliability of BPNN based on original data [22,23]. After comparing whale optimization algorithm (WOA), bacterial foraging optimization (BFO), particle swarm optimization (PSO) and genetic algorithm (GA), Tair et al. found that WOA algorithm has the best performance [24]. Currently, WOA has proven to be robust enough to find a good enough solution in a reasonable amount of time [25]. In addition, compared with the existing algorithms, WOA shows better or equivalent performance [26]. But the initial population position and the weight value in the shrinking mechanism of WOA will affect its performance. As one of the solutions, chaos theory is often used to improve the particle diversity of swarm intelligence algorithms [27]. RANI et al. used five chaotic maps (Chebyshev, Cubic, ICMIC, Neuron and Sine maps) to calculate the minimum values of eight functions (ShiftedSphere, Rastigin, Griewank, Exponential, Rosenbrock, Salamon, Csendes and Qing) [28]. The results show that the best performing chaotic mappings are Cubic and ICMIC. And Cubic outputs the minimum value with the smallest deviation error. Therefore, Cubic chaotic map is used as one of the methods to improve WOA. In addition, the adaptive weight adjustment strategy can be used to optimize the weight values in the shrinking boundary mechanism of WOA. This method can balance the early search ability and the later development ability of the algorithm [29]. In this paper, Cubic map and adaptive weight adjustment are used to optimize WOA. Therefore, the improved whale optimization algorithm (CA-WOA) is developed by the above two methods. Subsequently, the CA-WOA optimizes the weights and thresholds in BPNN, and the final CA-WOA-BPNN model is obtained. Finally, 60 debris flow data from Longmen Mountain are selected as a research sample in this study. This is because complex and inadequate sample data can be used to see whether the predictions are accurate and reliable.

The aim of this paper is to solve the problem of low accuracy and instability of BPNN under insufficient data and data complexity in the study of debris flow volume prediction. In Section 2, The relevant analysis, model construction process, comparative models, and performance indicators are introduced. In Section 3, the study area and models’ results are presented. In section 4, the content of this article and future work that needs further improvement are discussed in detail. In section 5, the conclusions of the CA-WOA-BPNN prediction model used in the research field of this article are introduced.

## 2 Method

### 2.1 Analysis of relationship

Correlation analysis is used to evaluate the correlation and importance between the influencing factors of the model, to select more relevant evaluation indicators. This paper uses Pearson correlation coefficient (PCC) and maximum information coefficient (MIC) for feature selection.

PCC is a well—known correlation measurement method with a range of [–1,1] [30]. Among them, 1 represents a completely positive correlation; -1 represents a completely negative correlation; 0 represents irrelevant. PCC as shown in Formula (1).


$$r=\frac{\sum_{i=1}^{n}\left(X_{i}-\bar{X})(Y_{i}-\bar{Y}\right)}{\sqrt{\sum_{i=1}^{n}{\left(X_{i}-\bar{X}\right)}^{2}}\sqrt{\sum_{i=1}^{n}{\left(Y_{i}-\bar{Y}\right)}^{2}}}$$ (1)

Where *X*_*i*_ and *Y*_*i*_ are the samples, and $\bar{X}$ and $\bar{Y}$ are the average values of the samples.

PCC as a linear analysis method, the factors with low correlation coefficient will not be effectively identified. However, the prediction accuracy of BPNN may be closely related to the factors with low correlation coefficient. The Maximum Information Coefficient (MIC) can be used to overcome this problem, which covers all the functional relationships. The prediction accuracy of the model is improved by combining MIC and PCC for feature selection. Mutual information (MI) is a measure of mutual trust between variables in information theory, as shown in Formula (2).


$$I(X;Y)=\int p(x,y){log}_{2}\frac{p(x,y)}{p(x)p(y)}dxdy$$ (2)

Where *p*(*x*, *y*) is the joint distribution of (*X*, *Y*), *p*(*x*) and *p*(*y*) are the marginal distribution.

The maximum information coefficient (MIC) is shown in Formula (3).


$$MIC(X;Y)=\underset{a\times b<B}{\max}\frac{I(X;Y)}{\log_{2}\left(\min\left(a,b\right)\right)}$$ (3)

Where *a* and *b* are the number of mesh partitions; *B* is the upper limit of the grid, usually *B* = *n*^0.6^, *n* is the number of samples.

### 2.2 Back Propagation Neural Network (BPNN)

#### 2.2.1 Standard BPNN

**Fig 1:**
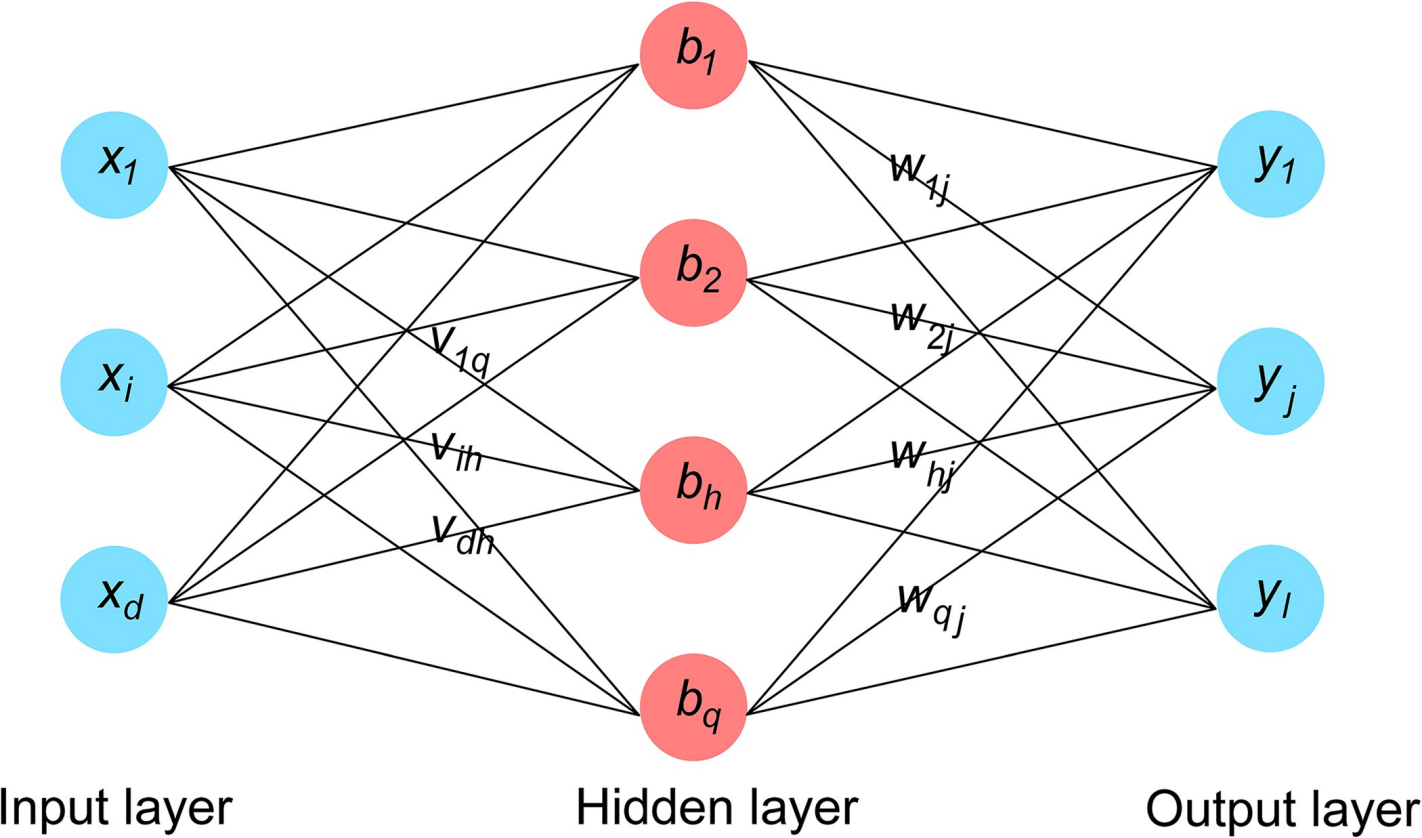
BP neural network structure diagram.

The BPNN is a feed-forward network trained by error back propagation algorithm, and it is also the most widely used network at present [31]. The network is composed of input layer, hidden layer and output layer. The weights between the layers are obtained by the forward propagation of the signal and the back propagation of the error. Then the BPNN model is established. The BPNN used in this paper is a single hidden layer network, as shown in Fig 1. It contains *d* input neurons *x*; *q* hidden layer neurons *b*; *l* output neurons *y*.

In the Fig 1, *v*_*ih*_ is the connection weight between the input layer neuron *x*_*i*_ and the hidden layer neuron *b*_*n*_; *w*_*hj*_ is the connection weight between the hidden layer neuron *b*_*n*_ and the output layer *y*_*i*_.

The input received by the hidden layer neuron *b*_*n*_ is shown in Formula (4).


$$\alpha_{h}=\sum_{i=1}^{d}v_{ih}x_{i}$$ (4)

The input received by the output layer neuron *y*_*i*_ is shown in Formula (5).


$$\beta_{j}=\sum_{h=1}^{q}w_{\mathrm{hj}}b_{h}$$ (5)

The training sample error is denoted as *E*_*k*_. Gradient descent is used as the adjustment strategy of neural network. Adjust the parameters in the negative gradient direction of the target. The learning rate *η* is assigned to the error *E*_*k*_, and the weight value is adjusted, as shown in Formulas (6) and (7).


$$\Delta w_{\mathrm{hj}}^{k}=-\eta •\partial E_{k}/\partial w_{hj}^{k}$$ (6)

$$w_{\mathrm{hj}}^{k+1}=w_{\mathrm{hj}}^{k}+\Delta w_{\mathrm{hj}}^{k}$$ (7)

The goal of the algorithm training is to make the cumulative error *E* of the training set meet the training accuracy requirements, as shown in Formula (8).


$$E=\frac{1}{m}\sum_{k=1}^{m}E_{k}$$ (8)

On the one hand, the oscillation of the network will be affected by the training rate in the neural network. On the other hand, the training rate is proportional to the convergence rate. The allowable error is generally set to 0.001–0. 00001.When the result error of more than two iterations is less than allowable error, the neural network iteration ends. In addition, the number of trainings is usually set to 1000 times. In summary, the key hyperparameters of BPNN in this paper are determined. The allowable error value is 0.000001; the training rate is 0.01; the number of trainings is 1000 times.

#### 2.2.2 Best hidden layer node

The selection of the number of hidden layers in neural networks has always been a hot topic in neural network research [32]. Too many hidden layer nodes will lead to an increase in training time and too much training [33]. On the contrary, too few hidden layer nodes will lead to insufficient network performance due to the complexity of data [33]. In this paper, the cycle method is used to determine the number of hidden layer nodes and training error. When initializing the setting, the mean square error (MSE) is set to a larger number. This step can find the minimum error and the best hidden layer node in the loop. The calculation of hidden layer nodes is shown in Formula (9) [34].


$$h=\sqrt{m+n}+a$$ (9)

Where *h* is the number of hidden layer nodes; *m* is the number of input layer nodes; *n* is the number of output layer nodes; *a* is generally an integer between 1 and 10.

### 2.3 Whale Optimization Algorithm (WOA)

**Fig 2:**
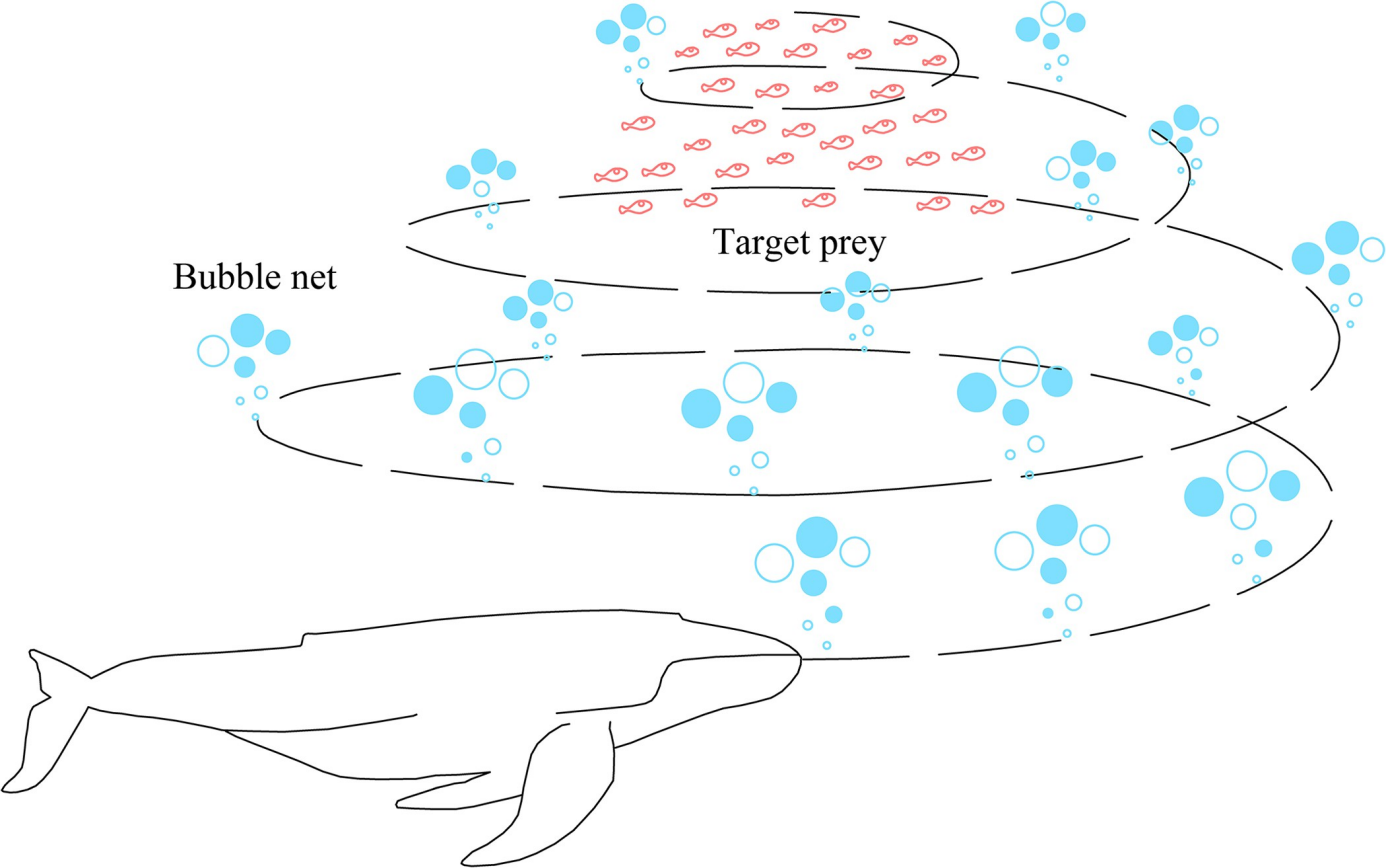
Bubble-net hunting behavior.

As a meta-heuristic optimization algorithm, WOA is mainly inspired by humpback whale hunting behavior [25]. Humpback whales have a special way of preying, known as bubble-net predation. The standard WOA simulates the unique search method and hunting mechanism of humpback whales. Its three important stages mainly include hunting prey, bubble net predation and searching prey. In WOA, each solution is considered as a whale. Whales try to fill new places in the search space, which is considered a reference to the best elements in the group. WOA searches the optimal solution in the solution space iteratively to find the best parameter configuration. In each iteration, WOA adjusts the weight and bias values according to the current solution space position and fitness value. The humpback whale foam hunting model is shown in Fig 2 [35].

#### 2.3.1 Surround prey

When the whale is hunting, it is necessary to find the location of the prey in advance when the prey is surrounded. But the location is usually unknown. Therefore, the WOA algorithm assumes that the solution with the smallest individual fitness value in the population is the position of the prey or the nearest position to the prey. Then, the positions of other search individuals are updated according to the optimal solution. The formula is as follows:

$$X(t+1)=X^{*}(t)-A\cdot D$$ (10)

Where *X* is the position vector of the search body; X* is the position vector of the current optimal solution; *A* is used to adjust the global exploration and local search ability of the algorithm; *D* is the distance vector between the current optimal solution and the search body. The calculation formulas of the *A* and *D* are as follows:

A=2ar−a (11)

$$D=|C\cdot X^{*}-X|$$ (12)

Where *a* decreases linearly from 2 to 0 with the number of iterations. The formula is as follows:

$$a=2\left(1-\frac{t}{T}\right)$$ (13)

Where *t* is the current number of iterations; *T* is the maximum number of iterations; *r* is a random vector in [0,1]; *C* is the coefficient vector. The formula is as follows:

C=2r (14)

#### 2.3.2 Bubble net hunting

WOA includes two position update strategies, namely the shrink-wrapping mechanism and the spiral position update. Among them, the shrink-wrapping mechanism is realized by the value of the convergence factor a in Formula (11). When |*A*| ≤ 1, the individual of the updated position moves from the original position to the target position. At this time, the shrink-wrapping mechanism is implemented by Formula (10). The spiral position update is to simulate the spiral motion state of the whale, as shown in Formula (15).


$$X(t+1)=D_{p}^{\prime }\cdot e^{bt}\cdot \cos\left(2\pi l\right)+X_{p}(t)$$ (15)

Where *b* is a constant used to limit the logarithmic spiral shape; *l* is a random number between [–1,1]; $D_{p}^{\prime }=|X*(t)-X(t)|$

Since the position of the whale is spirally updated, the encirclement is also reduced. The above synchronization process is realized by the probability *p* (*p* is a random number between [0,1]) in the WOA algorithm. This step is used to determine whether to perform the shrink-wrapping mechanism or the spiral position update. The formula is:

$$X(t+1)\left\{ \begin{array}{c} X^{*}(t)-A\cdot D\;p<0.5\\ D_{p}^{\prime }\cdot e^{bt}\cdot \cos(2\pi l)+X^{*}(t)\;p\geq 0.5 \end{array}\right.$$ (16)

#### 2.3.3 Prey search

When |*A*| > 1, the positions of other individuals are no longer updated by the target position in WOA. Instead, the position of the search body in the group is randomly found to replace the target position. This will enhance the global search ability of the algorithm. The update formula is as follows:

$$D=|C\cdot X_{\mathrm{rand}}-X|$$ (17)

$$X(t+1)=X_{\mathrm{rand}}-A\cdot D$$ (18)

Where *X*_*rand*_ represents the location of a random searcher in the current population.

In this paper, the initial population size of WOA is set to 30; the maximum number of iterations is set to 50; the independent variable interval is set to [–3,3]; the value of *b* in Formula (15) is set to 1.

### 2.4 Cubic map

The randomly generated initial population may cause WOA to fall into local optimum [36]. Because the algorithm group lacks diversity. In this study, Cubic map is added to the initial population position of WOA algorithm to optimize the randomly generated initial population position. Compared with the original random search, it can search the search space thoroughly with faster speed and probability. The Cubic map is shown in Formula (19).


$$x_{k+1}=\rho (1-x_{k}^{2})$$ (19)

Where *ρ* is the control parameter, *x*_*k*_ is the initial population generation method for WOA.

In this study, the value of *ρ* is 1.

### 2.5 Adaptive weight adjustment

In this study, an index-based adaptive weight adjustment method is proposed to optimize the weight value in the shrink-wrapping mechanism in WOA. The weight value changes with the number of iterations. To improve the weight value in the contraction closure mechanism in WOA. Adaptive weight adjustment is shown in Formula (20).


$$\omega =\omega_{min}+(\omega_{max}-\omega_{min})∙m_{m}exp(-t/maxgen)$$ (20)

Where *ω*_*min*_ is the minimum weight value; *ω*_*max*_ is the maximum weight value; *m*_*m*_ is the adjustment coefficient; *t* is the current number of iterations; max*gen* is the number of iterations; (−*t*/max*gen*) decreases with the increase of the number of iterations. In this study, the value of *m*_*m*_ is 1.

**Fig 3:**
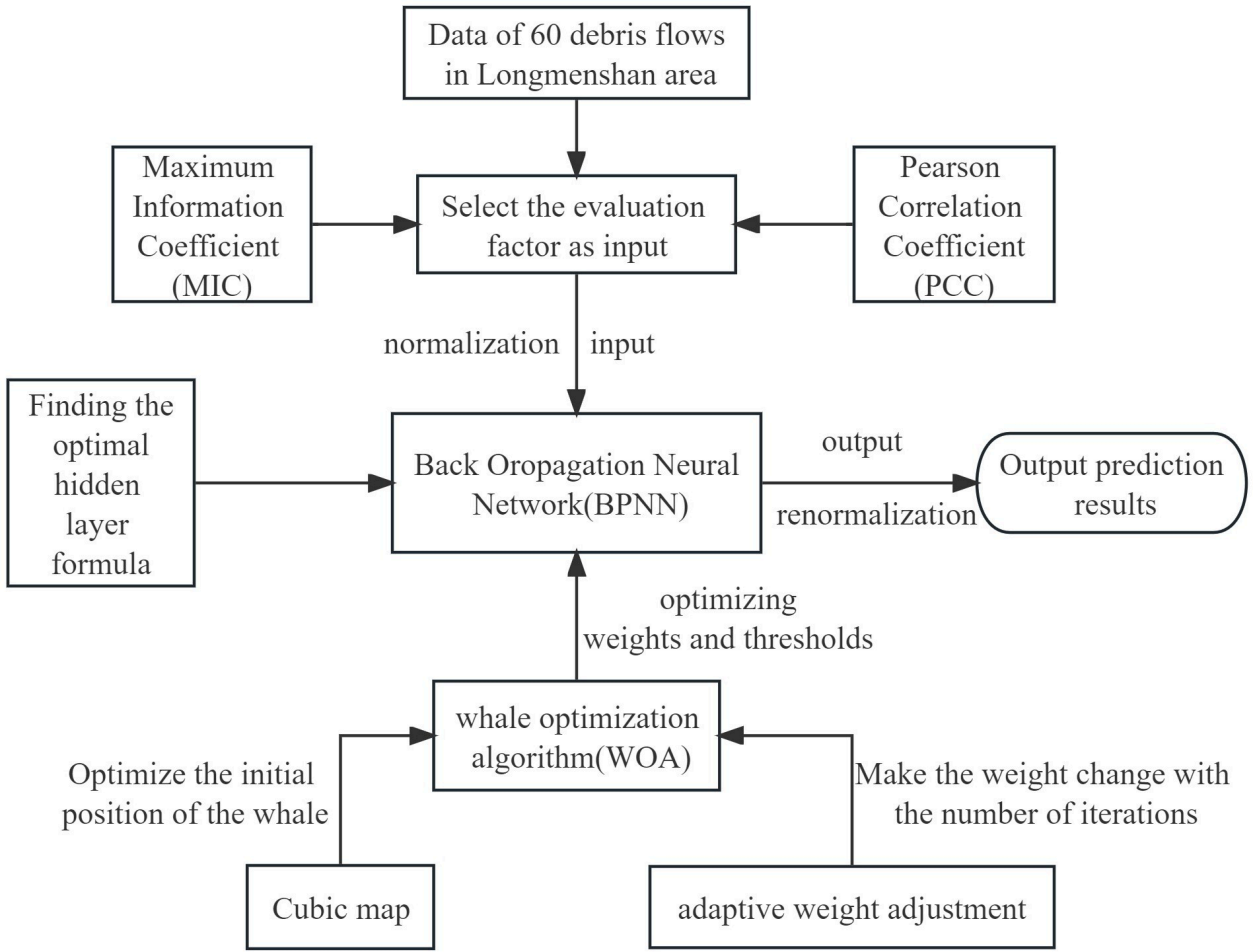
CA-WOA-BPNN model.

Firstly, Pearson correlation coefficient (PCC) and maximum information coefficient (MIC) are used to screen influencing factors to improve model accuracy, and then the data is normalized. Subsequently, Cubic map optimizes the initial population position in WOA. At the same time, the adaptive weight adjustment optimizes the weight value in the shrink-wrap mechanism in WOA. Finally, the improved WOA optimizes the weights and thresholds in BPNN to obtain the final CA-WOA-BPNN model. The whole workflow is shown in Fig 3, and the pseudo-code of the final model is shown in S1 Appendix.

### 2.6 Contrast model

To compare the prediction accuracy and stability of the final model, other machine learning algorithms need to be introduced for comparison [37]. SVR and XGBoost have the advantages of less time-consuming and high precision when the samples are not sufficient. Therefore, these two algorithms are applicable to the study area of this paper. Based on the Optuna framework, the key hyperparameters of SVR and XGBoost are adjusted. Optuna is an automatic hyper-parameter adjustment framework, including grid search method, random search method, Bayesian optimization algorithm and so on. [38]. The Bayesian optimization method is an effective optimization algorithm, which has been widely used in parameter optimization, hyper-parameter optimization and other fields [39].

In SVR, *c* is the penalty coefficient, which is understood as the tolerance of error. The *gamma* is a parameter attached to the selection of RBF function as kernel function. It implicitly determines the data distribution mapped to the new feature space.

In XGBoost, *n _ estimators* are the number of basic learners. The larger the number, the stronger the learning ability of the model, but the easier the model is to over-fit. The *max _ depth* is the depth of the tree and is an important pruning parameter. The *learning _ rate* is the step size of the iterative decision tree, also known as the learning rate. It controls the iteration rate of the algorithm and is often used to prevent overfitting. The *gamma* is used as a threshold to determine whether a leaf node should be further segmented.

On the other hand, to compare the performance of WOA and improved WOA, other meta-heuristic algorithms are selected for comparison. Artificial bee colony (ABC) and grey wolf optimization (GWO) are meta-heuristic algorithms that simulate the hunting behavior of other organisms in nature [40,41]. In addition, these two methods have been widely used in various optimization algorithm fields [42–45]. Therefore, this paper chooses ABC and GWO as comparative optimization algorithms to observe the performance of this method. The parameters of these two meta-heuristic algorithms are the same as those of the WOA algorithm in this paper (see Section 1.3 for details).

The key hyper-parameters of SVR, XGBoost, ABC and GWO are shown in Table 2.

**Table 2:** Key hyper-parameters.

Models	hyper-parameters
SVR	*c*: (1,1000) *gamma*: (0.001,1)
XGBoost	*n_estimators*: (100,2000) *learning_rate*: (0.01,1) *max_depth*: (1,10) *gamma*: (0.01,1)
ABC	*population size*: (30) *Maximum number of iterations*: (50) *Upper and lower limits of objective function*: (-3,3)
GWO	*population size*: (30) *Maximum number of iterations*: (50) *Upper and lower limits of objective function*: (-3,3)

### 2.7 Performance metrics

The Mean Absolute Error (MAE), Mean Absolute Percentage Error (MAPE) and coefficient of determination (R^2^) are used to evaluate the model. The smaller the MAE and MAPE, and the closer the R^2^ is to 1, the performance of the model is excellent. The MAE, MAPE, and R^2^ are shown in Formulas (21), (22), and (23).


$$MAE=\frac{1}{n}\sum_{i=1}^{n}|A_{t}-F_{t}|$$ (21)

$$MAPE=\frac{1}{n}\sum_{i=1}^{n}\left|\frac{A_{t}-F_{t}}{A_{t}}\right|$$ (22)

1. Where *A*_*t*_ is the real value of the sample; *F*_*t*_ is the predicted value of the model.

$$R^{2}=\frac{SSR}{SST}$$ (23)

Where *SSR* is the sum of regression squares; *SST* = *SSR*+*SSE*; *SSE* is the sum of residual squares. The calculations of *SSR* and *SSE* are shown in Formulas (24) and (25).


$$SSR={\sum \left(\hat{y}-\bar{y}\right)}^{2}$$ (24)

$$SSE={\sum \left(\hat{y}-y\right)}^{2}$$ (25)

Where $\hat{y}$ is the predicted value; $\bar{y}$ is the average value of the true value; and *y* is the true value.

Variance and standard deviation can reflect the degree of dispersion between data. Therefore, it is necessary to evaluate the final optimization function of each meta-heuristic algorithm (the weight value and threshold of BPNN in this paper). Calculate the variance (*S*) and standard deviation (*S*^2^) of the W_1_, W_2_ and B_1_. Among them, W_1_ is the weight value from the input layer to the middle layer; W_2_ is the weight value from the middle layer to the output layer; B_1_ is the neuron threshold of the middle layer. The calculation formulas of standard deviation (*S*) and variance (*S*^2^) are shown in Formulas (26) and (27).


$$S=\sqrt{\frac{1}{n}[{\left(x_{1}-\overset{-}{x}\right)}^{2}+{\left(x_{2}-\overset{-}{x}\right)}^{2}+\cdots +{\left(x_{n}-\overset{-}{x}\right)}^{2}]}$$ (26)

$$S^{2}=\frac{1}{n}[{\left(x_{1}-\overset{-}{x}\right)}^{2}+{\left(x_{2}-\overset{-}{x}\right)}^{2}+\cdots +{\left(x_{n}-\overset{-}{x}\right)}^{2}]$$ (27)

Where *n* is how many values are generated in each independent operation; $\overset{-}{x}$ is the average value.

## 3 Cases and results

### 3.1 Research area

The Longmenshan Mountain is located in a steep mountain range on the edge of the Qinghai-Tibet Plateau in southwestern China. The Longmenshan fault zone starts from the Luding area in the southwest and passes through Wenchuan, Guanxian, Beichuan, Yangpingguan, and Mianxian in the northeast. It passes through the Qinling Mountains for more than 600 kilometers. The Wenchuan earthquake occurred on the Longmenshan fault zone. This makes the Longmenshan fault zone become one of the key research areas [46].The debris flow in the study area is distributed in the rugged mountains of 407 ~ 6100 m. The slope direction is basically along the northeast direction, the slope is as high as 69°, and more than half of the slope is greater than 36° [47].

The rocks in the mountain area are mainly composed of basalt, granite, phyllite, dolomite, limestone, sandstone and shale. The age is from Precambrian to Cretaceous, with strong fracture and weathering characteristics [46]. In addition, in the above geomorphological features, it is easy to produce large thrust earthquakes with coseismic landslip [48].

The loose material produced by the earthquake is prone to debris flow disaster under the action of heavy rainfall or ice and snow melting. Therefore, after the Wenchuan earthquake, landslides or debris flow events occur every monsoon season. Previous studies have shown that under the action of earthquakes, debris flow events will occur many times in the catchment area of the study area [46].Therefore, it is necessary to make predictions about the region.

In other debris flow volume prediction studies, catchment area (A), topographic relief (H), channel length (L), and average channel gradient (J) are used as the main factors [49,50]. The study area in this paper is on the seismic zone. A large number of loose materials produced by the Wenchuan earthquake in 2008 provided excellent source conditions for debris flows. Therefore, total volume of co-seismic landslide debris (V) and distance from seis-mic fault (D) are added to the research factors in this paper [46]. According to the above six influencing factors, sixty typical debris flow data in this area are selected, as shown in Table 3 [46].

**Table 3:** Basic data of sixty debris flows.

No.	A(km^2^)	H(m)	L(km)	D(km)	J (‰)	V(10^4^m^3^)	V_0_(10^4^m^3^)
1	14.74	882	4.56	1.46	193.42	485.76	115.11
2	0.69	565	1.93	2.87	292.75	49.53	10.68
3	1.54	374	2.22	3.19	168.47	25.58	6.2
4	0.65	180	1.45	0.57	124.14	7.01	2.8
5	8.17	1150	3.94	0.06	291.88	1603.25	285.39
6	8.91	970	3.1	4.42	312.9	920.7	213.13
7	3.83	640	2.41	0.88	265.56	195.9	58.81
8	0.5	470	1.2	2.08	391.67	42.84	8.24
9	3.25	920	2.61	2.54	352.49	996.46	182.44
10	8.63	1605	4.45	1.49	360.67	858.94	250.06
11	0.1	188	0.58	1.44	324.14	11.6	2.82
12	2.22	754	1.43	0.85	527.27	375.7	65.23
13	0.72	531	0.74	0.46	717.57	35.1	7.86
14	9.8	1162	4.51	9.58	257.65	1754.64	162.64
15	30.84	910	4.01	3.42	226.93	2000	800
16	2.17	1000	1.15	2.52	869.57	27.66	12.32
17	3.05	698	2.17	1.6	321.66	299.21	69.92
18	0.86	972	1.46	8.86	665.75	64.56	18.12
19	2.97	1027	1.12	10.16	916.96	121.23	66.35
20	3.66	865	1.26	5.3	686.51	592.74	166.35
21	0.74	529	0.78	2.5	678.21	112.51	8.56
22	4.06	772	2.8	5.5	275.71	405.1	104.38
23	0.72	643	0.68	0.26	945.59	45.36	6.35
24	0.86	585	0.74	1.55	790.54	34.51	5.34
25	0.53	463	0.67	1.29	691.04	29.84	4.31
26	0.62	532	0.84	0.17	633.33	35.21	5.38
27	0.69	546	0.74	0.57	737.84	30.64	4.71
28	7.81	1490	4.9	3.6	304.08	1580.2	657.3
29	1.36	1177	2.59	0.87	454.44	334.3	156.8
30	5.72	986	3.66	0.16	269.4	432.66	108.61
31	8.43	963	4.02	3.8	239.55	964.86	292.1
32	5.24	876	2.08	7.6	421.15	234.64	51.42
33	6.51	886	4.98	10.5	177.91	287.52	67.93
34	6.27	1870	5.6	16.89	333.93	358.26	74.12
35	5.35	1288	3.6	0.13	357.78	358.14	98.4
36	10.7	1842	5.82	0.42	316.49	1151.41	218.72
37	2.18	1820	2.72	0.72	669.12	322.3	80.4
38	54.2	2900	14.2	0.47	204.23	2180.57	505.34
39	0.06	984	1.58	0.65	622.78	222	77.3
40	7.5	935	5.2	4.39	179.81	647.48	150.5
41	2.18	1220	2.68	4.93	455.22	122.3	92.51
42	5.21	1678	3.4	2.51	493.53	321.32	89.16
43	1.21	1596	1.14	2.07	140	13.8	3.62
44	10.39	1453	5.51	2.61	263.7	727.04	108.91
45	16.49	2382	6.2	0.68	384.19	742.68	194.52
46	1.12	1000	3.25	1.36	307.69	35.93	8.21
47	0.97	1600	1.87	2.1	853.33	48.84	15.3
48	0.29	1580	2.1	1.97	752.38	17.22	4.38
49	21.7	2952	8.9	0.57	331.69	366.67	136.07
50	0.46	650	1.3	1.68	500	31.63	8.97
51	1.98	965	2.44	0.92	395.29	81.4	28.89
52	1.54	1002	2.35	1.53	426.38	77.6	16.3
53	0.68	952	1.95	1.71	488.21	191.29	46.67
54	8.32	1668	4.76	3.86	350.42	136.02	29.25
55	0.2	434	1.21	0.91	358.68	21.46	6.29
56	0.21	440	1.26	1.82	349.21	19.17	2.54
57	0.29	460	1.35	0.85	340.74	26.13	7.94
58	0.64	660	1.98	4.8	333.33	59.9	15.6
59	1.55	1120	4.01	0.45	279.3	270.16	94.5
60	36.77	1203	11.92	7.04	100.92	1200.26	311.93

In the above table, the A is catchment area, the H is topographic relief, the L is channel length, the D is distance from seismic fault, the J is average channel gradient, the V is total volume of co-seismic landslide debris, and the V_0_ is the volume of debris flow.

### 3.2 Data processing

#### 3.2.1 The normalized processing

The sixty debris flow evaluation factors in Table 2 are taken as the decision variable matrix Z, where Z = {*z*_*ij*_} (*i* = 1, 2…, *m*; *j* = 1, 2…, *n*). The *m* represents the number of debris flow, *m* = 60; *n* represents the number of evaluation factors, *n* = 6. Due to the different dimensions of each evaluation factor, the results will be affected, and the decision matrix needs to be normalized. The evaluation factors are normalized using the minimum-maximum and mapped to the [0, 1] interval, the normalization as shown in Formula (28).


$$\tilde{z_{\mathrm{ij}}}=\frac{z_{\mathrm{ij}}-min(z_{1:m,j})}{\max\left(z_{1:m,j}\right)-min(z_{1:m,j})}$$ (28)

Where max () represents the maximum value; min () represents the minimum value.

#### 3.2.2 Analysis of relationship

**Fig 4:**
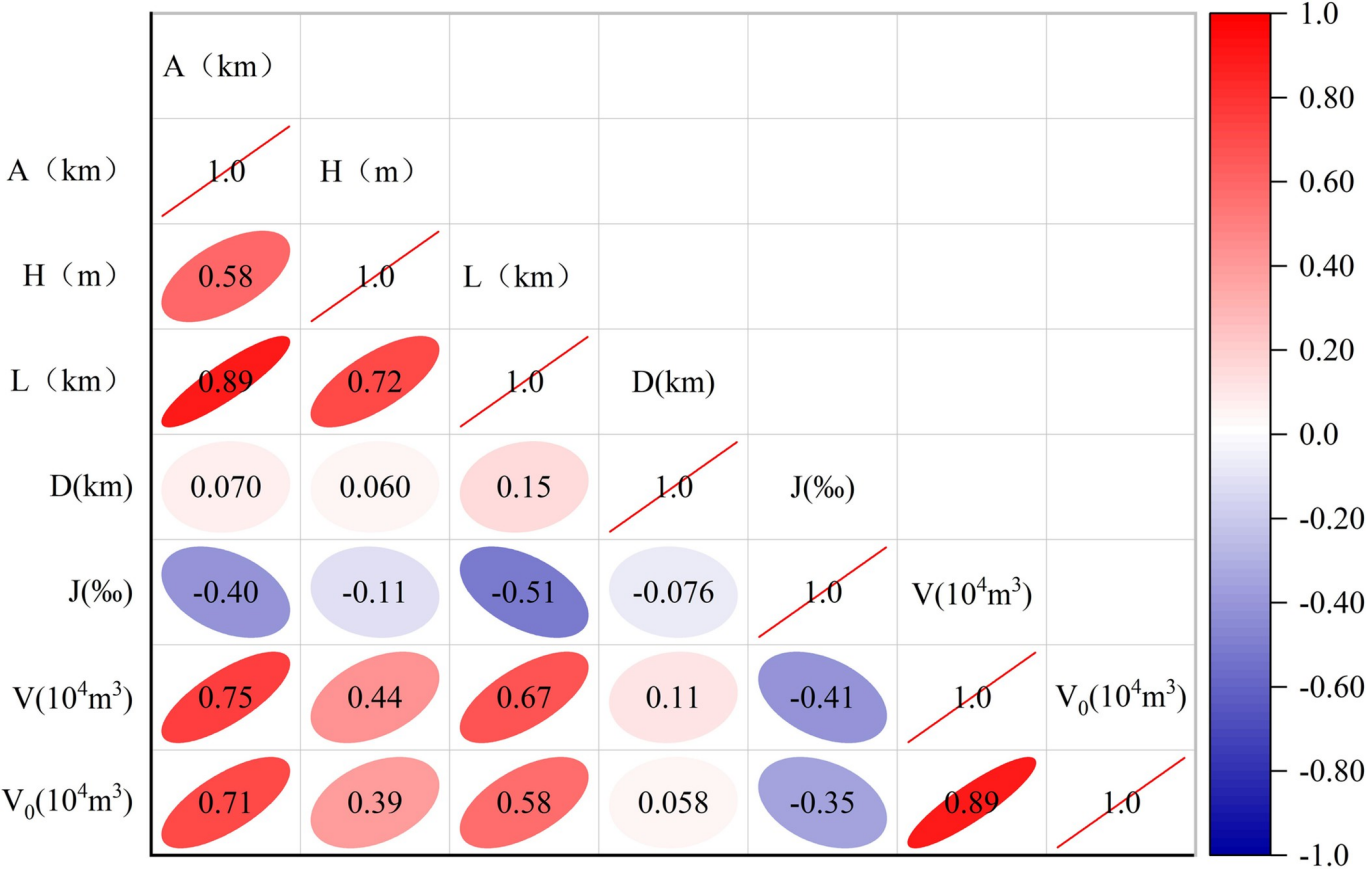
Correlation analysis of each influencing factor. The left longitudinal axis and the symmetrical line are the influencing factors, and the redder the color, the greater the correlation.

PCC and MIC are used to analyze the correlation between the basic data in Table 3. A, H, L, D, J, V and V0 are then sorted according to their importance. The results are shown in Fig 4 and Table 4.

**Table 4:** The importance between 6 factors and V_0_.

code	PCC	MIC	Ranking of importance
V	0.89	0.97	1
A	0.71	0.82	2
L	0.58	0.69	3
H	0.39	0.63	4
J	-0.35	0.34	5
D	0.06	0.25	6

The above results show that the PCC of V and V0 is 0.89, which is the largest positive correlation coefficient. The PCC of other factors are A and V_0_ (0.71), L and V_0_ (0.58), H and V_0_ (0.39), D and V_0_ (0.06) and J and V_0_ (-0.35), respectively. In addition, the MICs of D and J are only 0.25 and 0.34, respectively.

Therefore, combined with the results of PCC and MIC, only V, A, L and H are selected as input factors to improve the prediction accuracy of the model. The input factors ratio is 8:2, that is, forty-eight as the training set and twelve data as the prediction set.

### 3.3 Model prediction results and optimization algorithm analysis

#### 3.3.1 Model prediction results

**Fig 5:**
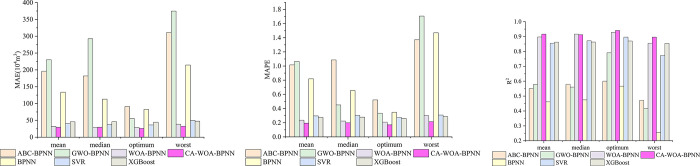
The evaluation results of each model after five independent operations.

Each model is run independently for 5 times, and each prediction result is brought into Formulas (21)–(23) for 5 calculations. Subsequently, the average, median, optimal and worst values are used to calculate the results of five calculations to represent the prediction accuracy and stability of the model, as shown in Fig 5. In addition, the training set and prediction set of each model are the same.

In Fig 5, the smaller the MAE and MAPE, the larger the R^2^, and the better the prediction performance of the model.

From the two metrics of MAE and MAPE, the prediction accuracy of CA-WOA-BPNN model is the highest, followed by WOA-BPNN. Then there are SVR and XGBoost, and the performance of these two models is almost the same. The worst are ABC-BPNN, GWO-BPNN and BPNN. It is worth noting that the MAE and MAPE of ABC-BPNN and GWO-BPNN are larger than those of BPNN.

From the fitting degree (R^2^) of the model, the model fitting degree of CA-WOA-BPNN is also the best, followed by WOA-BPNN. Then there are SVR and XGBoost, and the R^2^ of these two models is almost the same. In addition, the R^2^ of ABC-BPNN and GWO-BPNN is better than that of BPNN, which is different from the evaluation results of MAE and MAPE.

In general, the model (CA-WOA-BPNN) in this paper has the highest prediction accuracy and the best fitting degree.

#### 3.3.2 Objective function results of meta-heuristic algorithm

**Fig 6:**
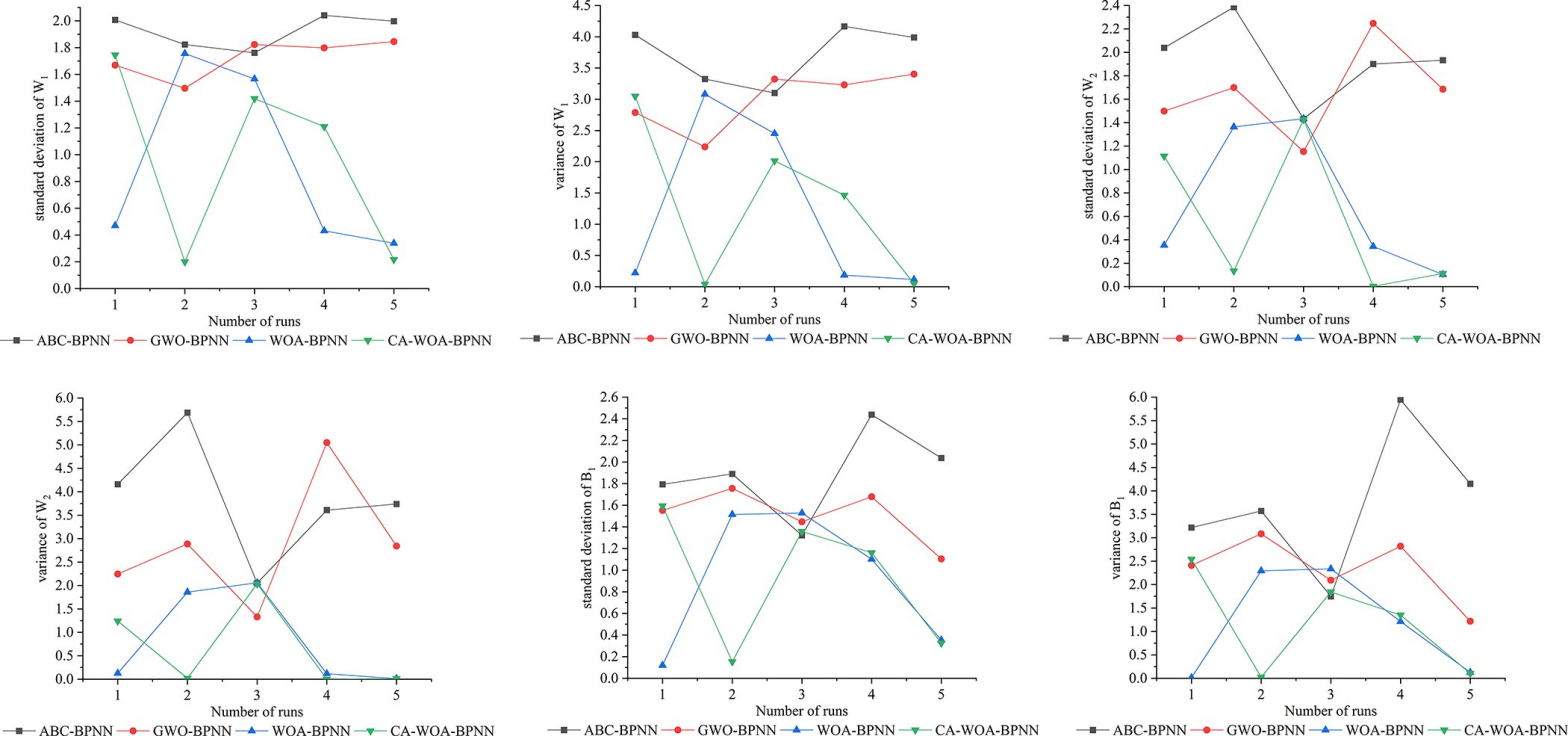
Variance and standard deviation of W_1_, W_2_ and B_1_. In Fig 6, the left side is the standard deviation, the right side is the variance, the vertical axis is the value; and the horizontal axis is the number of runs.

The standard deviation and variance of the objective function (W_1_, W_2_ and B_1_) output by the four meta-heuristic algorithms (ABC, GWO, WOA and CA-WOA) are shown in Fig 6. Among them, the neural network parameters in ABC-BPNN, GWO-BPNN and WOA-BPNN are the same, and the hidden layer is set to 9. Different from the above three algorithms, CA-WOA-BPNN is set to automatically find the number of hidden layers. Moreover, the search interval of all the 4 meta-heuristic algorithms is [–3,3].

The greater the standard deviation and variance, the greater the degree of dispersion between the data. In Fig 6, the variance and standard deviation of ABC-BPNN and GWO-BPNN are almost greater than those of WOA-BPNN and CA-WOA-BPNN. This shows that the dispersion between the weight value and the threshold calculated by WOA-BPNN and CA-WOA-BPNN is smaller. In addition, the results in Fig 5 show that the prediction performance of WOA-BPNN and CA-WOA-BPNN is better than that of ABC-BPNN and GWO-BPNN. Combined with the results in Fig 6, the optimization performance of ABC and GWO is weaker than that of WOA and CA-WOA under the same search interval and the data in this paper.

The above results show that the prediction accuracy and reliability of CA-WOA-BPNN are better than the other six algorithms. Under the same parameter setting and data set, the optimization effect of WOA on neural network is obviously better than that of ABC and GWO. In addition, the improved WOA can find better weights and thresholds in BPNN. In general, the prediction results of CA-WOA-BPNN can provide reference for debris flow prevention and emergency rescue under insufficient data.

## 4 Discussion

Through correlation analysis, four main factors affecting the volume of debris flow in Longmenshan area are determined. They are co-seismic landslide debris (V), catchment area (A), channel length (L) and topographic relief (H). Among them, the total volume of co-seismic landslide debris has the greatest correlation with debris flow volume. Because the study area is in the seismic zone, the landslide material produced by the Wenchuan earthquake provides an excellent source condition for the debris flow. In addition, Fig 4 shows that the other three input factors (A, L and H) are also highly correlated with V. Therefore, it is necessary to start the preliminary work of predicting the volume of mudslides with a correlation analysis.

The Fig 5 shows that there is almost no difference between the mean, median, optimal and worst values of CA-WOA-BPNN, WOA-BPNN, XGBoost and SVR. This means that the prediction results of their four models are more stable. On the contrary, the prediction results of BPNN, GWO-BPNN and ABC-BPNN are unstable. In addition, the prediction performance of SVR and XGBoost is inferior to that of CA-WOA-BPNN. This shows that in the absence of data and complex data relationships, SVR and XGBoost need more learning samples for reference. Even though these two models have been proven to exhibit good predictive performance in the absence of data. On the contrary, the CA-WOA-BPNN prediction model proposed in this paper is more suitable for this research field. It has high accuracy and reliability in the absence of data and strong correlation between data. Especially in the prediction of debris flow volume, only high precision is often not enough. Good stability is also one of the criteria to be considered. Because only one low-precision prediction result may make the disaster relief personnel bear greater risks. Therefore, the CA-WOA-BPNN in this paper is more suitable for the study area.

**Fig 7:**
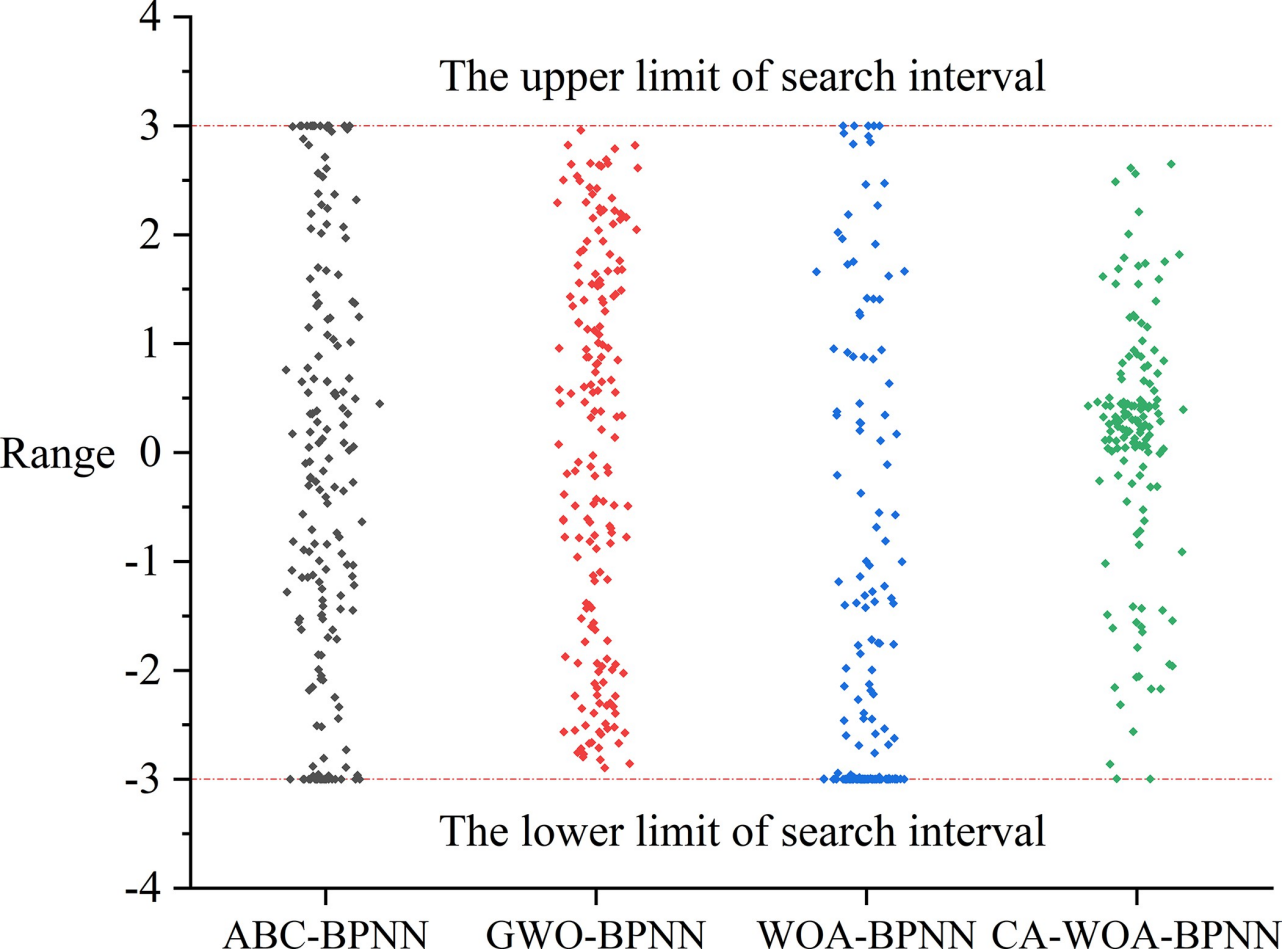
W_1_ output by meta-heuristic algorithms.

In Fig 6, the deviation between GWO and ABC output results is greater than that of CA-WOA and WOA. In order to find out the cause of this problem, all the output results of W_1_ are drawn, as shown in Fig 7. Fig 7 shows that the scatter points of ABC-BPNN and GWO-BPNN are basically evenly distributed between [–3,3]. On the contrary, the scatter points of WOA-BPNN are clustered at—3. The situation of CA-WOA-BPNN is similar to that of WOA-BPNN, and its scatter points are clustered between [0,1]. This is due to the optimization of WOA by Cubic map and adaptive weight adjustment strategy. At the same time, Fig 5 shows that the prediction performance of CA-WOA-BPNN is the best. This indicates that the weight and threshold of CA-WOA output are more suitable for the data set of this paper. This also proves that the optimization of Cubic map and adaptive weight adjustment strategy is effective.

**Fig 8:**
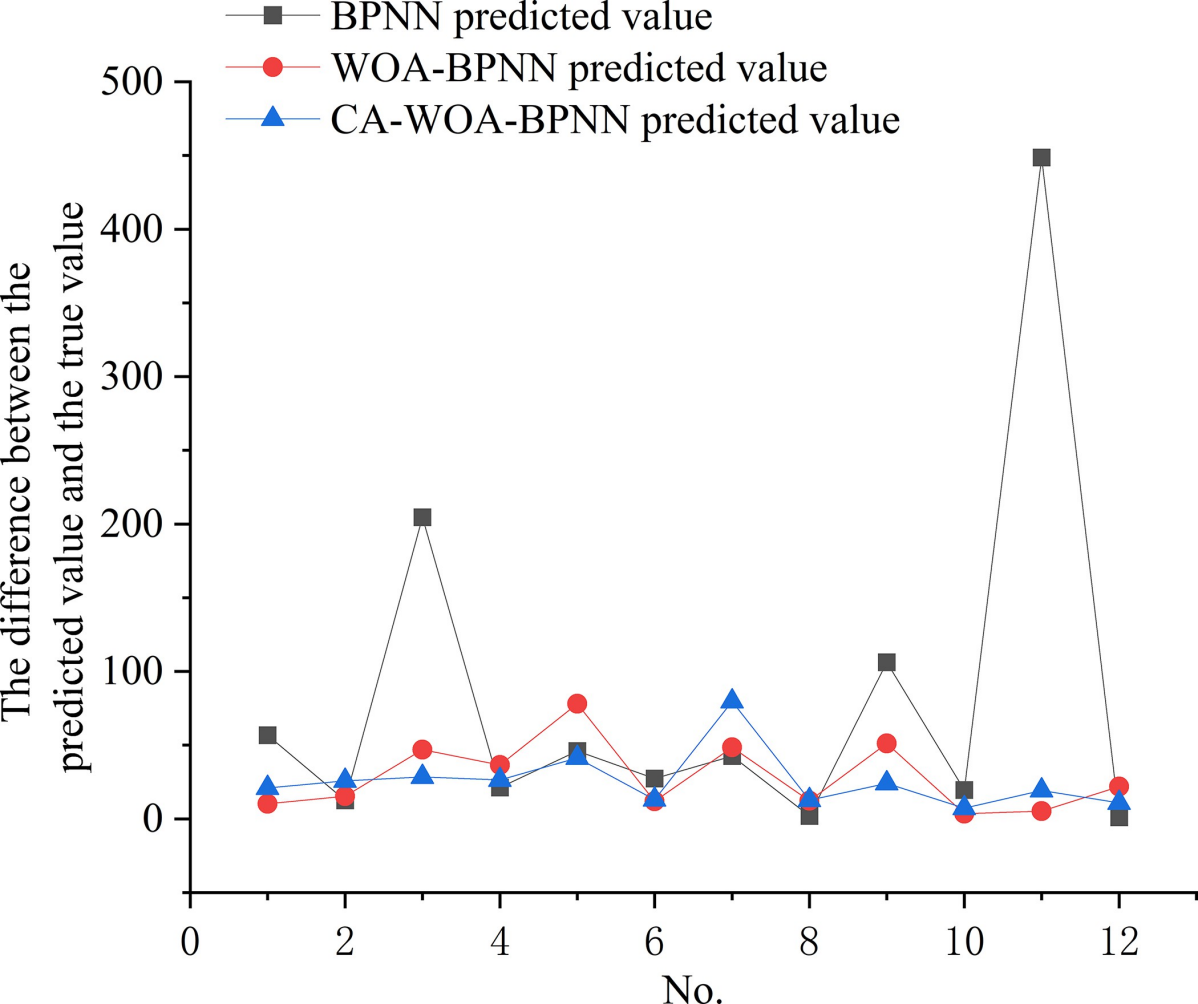
The difference between the predicted value and the true value of BPNN, WOA-BPNN and CA-WOA-BPNN.

The optimal prediction results of three models (BPNN, WOA-BPNN, CA-WOA-BPNN) are selected. Then the absolute values of the difference between the predicted value and the real value are used as the index to draw Fig 8. The Fig 8 shows that the errors of WOA-BPNN and CA-WOA-BPPN are much smaller than those of BPNN. Especially in the prediction of large debris flow volume (No.11). In addition, the maximum errors of BPNN, WOA-BPNN and CA-WOA-BPNN are NO.11, NO.5 and NO.7, respectively. These analyses show that the improved BPNN is superior to BPNN. Especially in the prediction of large volume debris flow. Moreover, CA-WOA-BPNN does not misjudge small-volume debris flows as large-volume. This is beneficial for disaster relief personnel to carry out rescue after debris flow disaster. Because the correct prediction results can provide a reliable reference for disaster relief.

In general, CA-WOA-BPNN can achieve high accuracy and stability under insufficient samples and complex data. Under the same parameter settings, the optimization effect of CA-WOA is better than that of WOA, ABC and GWO. This is benefit by Cubic map and adaptive weight adjustment strategy. Therefore, CA-WOA-BPNN overcomes the instability and low prediction accuracy of BPNN under insufficient and complex data. In other words, this method can provide a favorable reference for debris flow prevention and disaster relief. However, this paper still has some limitations:

1. The performance of machine learning algorithms is related to the quality of data sets. Therefore, how to improve the quality of data sets is the key to achieve efficient prediction. For example, using data simulation software generate data similar to the study area. Then, it is used as input for learning. Finally, the predicted value of the output is compared with the real value. This method can not only test whether the generated data is reliable, but also can expand the data set in a low-cost way.
2. As the data increases, the running time of the model will be longer. Therefore, it is necessary to focus on how to shorten the running time. Research shows that fuzzy logic can tolerate data inaccuracy and reduce running time [51]. In addition, it can also improve the generalization ability of neural networks [51]. Therefore, fuzzy logic is also a reference for further research.
3. The optimization results of ABC and GWO are unsatisfactory. Therefore, how to adjust the hyperparameters of these two algorithms needs to be further explored.

## 5 Conclusion

In this paper, the sixty debris flows in Longmenshan area are taken as research cases. Correlation analysis is used to identify the four main factors affecting the study area. Then, the Cubic map and adaptive weight adjustment are used to optimize the whale optimization algorithm (WOA). Subsequently, the improved WOA is used to optimize the Back Propagation Neural Network (BPNN). Finally, the CA-WOA-BPNN debris flow volume prediction model is established to carry out the study. The following conclusions are obtained:

1. In the Longmenshan area, co-seismic landslide debris is the main factor influencing the volume of debris flows. This is because loose material from earthquakes provides excellent source conditions for debris flows.
2. CA-WOA-BPNN outperforms the other six models in terms of reliability and stability. The model can overcome the instability of BPNN under insufficient and complex data. This is attributed to the optimization of WOA by Cubic map and adaptive weight adjustment strategies. In which, Cubic map optimizes the initial population position of WOA. In addition, adaptive weight adjustment optimizes the weight value of the shrink-wrap mechanism in WOA. Overall, CA-WOA-BPNN can provide an effective reference for debris flow volume prediction studies with insufficient samples and complex data.
3. In future studies, more attention needs to be paid to model runtime, dataset quality, and hyperparameter selection.

## Supporting information

S1 Dataset The minimum data set. (RAR)

S1 File Codes and related data. (RAR)

S1 Appendix (DOCX)
