# Supplementary material for: Debris flow volume prediction model based on back propagation neural network optimized by improved whale optimization algorithm
Source: PLoS One. 2024 Apr 9;19(4):e0297380. doi: 10.1371/journal.pone.0297380 (PMC11003783; doi:10.1371/journal.pone.0297380)
Supplement: S1 Appendix — (DOCX) [file pone.0297380.s003.docx]

**Appendix.1**

| **Algorithm 1: BP neural network for optimal hidden layer nodes** | |
| --- | --- |
| **Input:** | Catchment area (A)、Topographic relief (H)、Channel length(L) and Total volume of co-seismic landslide debris(V) |
| **Output:** | Debris flow volume (V_0_) |
| **Step 1:** | Normalize input and output data. |
| **Step 2:** | Set the number of input and output layer nodes. |
| **Step 3:** | Set the minimum mean square error (MSE). |
| **Step 4:** | **For** The optimal number of hidden layer nodes is determined by using formula (9). |
| **Step 5:** | Build a network and set network parameters for training. |
| **Step 6:** | Obtain simulation results and calculate the mean square error (mse) of the simulation. |
| **Step 7:** | **If** mse<MSE, MSE=mse, and the optimal hidden layer node=the current hidden layer node.  **End** |
| **Step 8** | **End** |
| **Step 9** | Establish the BP neural network with the best hidden layer nodes and set parameters for training. |
| **Algorithm 2: Improved Whale Optimization Algorithm** | |
| **Step 10** | Set the maximum population size *N* and the maximum number of iterations *T*. |
| **Step 11** | Set the leader position by using formula (19) and setting the leader score. |
| **Step 12** | Initialize the population and determine the optimal individual *X*^*^. |
| **Step 13** | **For** each individual, check if it exceeds the boundary. Exceeding the boundary value is replaced by the boundary value. |
| **Step 14** | Calculate the objective function value. |
| **Step 15** | **If** the objective function value < the leader's score, the leader's position=the current position. |
| **Step 16** | **End** |
| **Step 17** | **End** |
| **Step 18** | Calculate the *a* value for each individual by using formula (13). |
| **Step 19** | Calculate the *A* value for each individual by using formula (11). |
| **Step 20** | Calculate the *C* value for each individual by using formula (14). |
| **Step 21** | Set the weight values in the shrink wrap by using formula (18). |
| **Step 22** | **For** each individual. |
| **Step 23** | **If** p<0.5 |
| **Step 24** | **If** abs(A)>=1 |
| **Step 25** | Calculate the *D* value by using formula (17). |
| **Step 26** | Update the position by using formula (18). |
| **Step 26** | **Else if** abs(A)<1 |
| **Step 28** | Calculate the *D* value by using formula (12). |
| **Step 29** | Update the position by using formula (10). |
| **Step 30** | **End** |
| **Step 31** | **Else if** p>=0.5 |
| **Step 32** | Update the position by using formula (15). |
| **Step 33** | **End** |
| **Step 34** | **End** |
| **Step 35** | Output optimal value |
| **Algorithm 3: Improved BP neural network algorithm** | |
| **Step 36** | Update weight values and threshold values by leader position |
| **Step 37** | Constructing a New BP Neural Network |
| **Step 38** | Training with optimized BP neural network |
| **Step 39** | Using optimized BP neural network for prediction |
| **Step 40** | Output Results |
